# Riddle in Nine Syllables

**DOI:** 10.3201/eid1809.AC1809

**Published:** 2012-09

**Authors:** Polyxeni Potter

**Affiliations:** Centers for Disease Control and Prevention, Atlanta, Georgia, USA

**Keywords:** art science connection, emerging infectious diseases, art and medicine, Riddle in Nine Syllables, Paul Jacoulet, Le Trésor (Corée), ukiyo-e, maternal and child health, Sylvia Plath, Metaphors, about the cover

**Figure:**
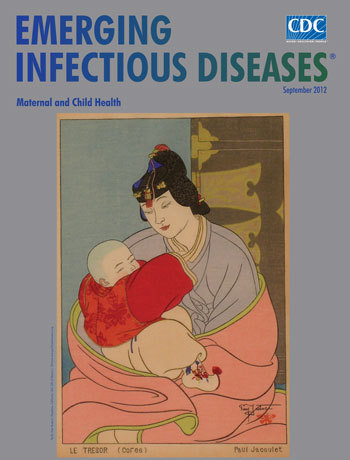
**Paul Jacoulet (1902–1960) *Le Trésor *(*Corée*) Japan, 20th century Ink and paper (overall 23.5 cm × 30.2 cm; image 14.6** **cm × 9.8 cm; card 14.6 cm × 9.8 cm)** Pacific Asia Museum, Pasadena, California, USA, Gift of Eleanor L. Gilmore www.pacificasiamuseum.org

“An elephant, a ponderous house / A melon strolling on two tendrils” is how Sylvia Plath described herself in “Metaphors.” In addressing her physical condition, the poet was acknowledging the psychological toll of changes to the female body during pregnancy, which she termed “a riddle in nine syllables.” The immensity of these changes has long preoccupied artists too, while the public health implications were left to physicians and others concerned with maternal and child health.

It was along these lines that artist Paul Jacoulet addressed the theme of maternity in *Le Trésor *(*Corée*) (The Treasure [Korea]), on this month’s cover: maternity as a stage of life. Like Japanese master printer and painter of women Kitagawa Utamaro (1753–1806), a formative influence, Jacoulet was interested in mother and child as a special population, depicting them in their daily activities as persons with individuality and character.

Jacoulet was born in Paris, a frail child, “like a damaged little worm,” ill with chronic bronchial problems. His family moved to Japan when he was very young. He grew up in Tokyo firmly grounded in Japanese culture, multilingual, with early training in music, dance, drama, and calligraphy. But art prevailed. “Almost before I spoke, I was glad to have a pencil in my hand.” He started to paint at age 11 under artist Seiki Kuroda (1883–1924), who taught Western art theories to the Japanese. Jacoulet grew up next door to *ukiyo-e* authority Yone Noguchi, father of sculptor Isamu Noguchi. Isamu’s mother, American writer Léonie Gilmour, taught Jacoulet English.

An avid student of *ukiyo-e*, the woodblock print genre, Jacoulet quickly mastered and abandoned it for a style, uniquely his own, which combined exacting technique and Japanese brushwork with Western influences (Paul Gauguin, Édouard Manet, Henri Matisse) from his frequent trips to Paris and from a personal approach to line drawing and color use. Jacoulet was an innovator. He introduced embossing for added texture and enriched his prints with colored micas, crushed pearls, lacquers, and silver, gold, and bronze metallic pigments for a shimmering sensuous effect.

“The woodcut colorprint is like music,” Jacoulet believed, “Without harmony among painter, engraver, and printer, it is impossible to produce a fine picture.” He engaged only distinguished carvers and printers and stamped their names on the margins of prints. He established the Jacoulet Institute of Prints and published nearly all his work himself, resisting massive production of copies. He brought only the best watermarked paper and boasted using as many as 300 blocks for one print. He produced thousands of drawings and water colors, many now lost; 166 color woodblock prints survive.

Instead of the usual young and beautiful theater performers and courtesans of traditional *ukiyo-e*, Jacoulet’s work featured the aging and weak whose faces he observed and recorded in sketches and photographs during his many travels, widely throughout Japan, China, Mongolia, and the South Pacific Islands. Of special interest were the indigenous people of these areas and the Western residents of Japan, where he lived and worked most of his life. A confirmed naturalist, he collected specimens and painted disappearing wildlife and small villages overrun by modern civilization, becoming at times the only chronicler of island populations now extinct. In this way, his work was imbued effortlessly with the exoticism so sought after on the Western art scene.

“I am the greatest artist,” Jacoulet wrote to collectors in the 1950s, seeking acceptance and recognition. His unconventional approach to painting and printing and unusual choices of subject matter did not make him popular. “I am anxious and rather down, very discouraged,” he wrote to friends who tried to expose his work to a broader audience. By then, the once described “best looking young man in Tokyo,” took to appearing in public with his face powdered and lips tinted with rouge, perhaps to correct a sallow complexion brought on by illness.

*Le Trésor* is from a series on Korean subjects. Jacoulet visited Korea frequently after the death of his father during War World II, when his mother moved there to live with her new husband, a Japanese physician. In addition to all manner of local characters, from scholars and the wealthy to common workers and beggars, he covered in this series of prints mothers with their children, a subject common in European as well as Japanese art. *Le Trésor* sold more than 300 copies.

The mother’s face is common but clearly focused, the tassel on her headpiece undone, exaggerating her downward look and leading the viewer to the center of the picture. Her body envelops the child, fluid circular lines making a nest for the red bundle. This tender, private moment is sparingly drawn, accented only with the bold vest and decorated footwear of the child, whose little hand is reaching inside the mother’s neckline.

Mother and child, one of the oldest and most frequent subjects in the history of art, draws on the universality of the complex psychological experience of having and being a mother. By the 19th century, religious and romanticized images of maternity gave way to a more down to earth approach, though the notion of mother as vessel without much control persisted. Like many artists of his generation, Jacoulet explored the uniqueness of the experience by capturing facets of maternity in the floating world, just as, in her own way, looking inward, Sylvia Plath examined changes in the context of a riddle.

Maternal and child health, its own riddle intertwined with pregnancy, features also in disease emergence because special populations, pregnant women among them, and their response to emergence are key to successful disease prevention and control. Jacoulet did not know and Sylvia Plath could only sense the physical hazards involved in being a vessel. The genetically foreign fetus challenges a woman’s core defense against disease, the immune system, which has to make changes if the pregnancy is to succeed. These changes, not well understood, may alter susceptibility to and severity of certain infectious diseases (toxoplasmosis, listeriosis, malaria, measles) and could increase death rates from others, such as influenza and varicella. Hepatitis E virus infections continue to cause a disproportionate number of deaths among pregnant women in developing countries, despite the availability of vaccines. These and other still unknown health threats add meaning to the poet’s lament. “I’m a mean, a stage, a cow in calf. / I’ve eaten a bag of green apples, / Boarded the train there’s no getting off.”
